# Evaluating the morphology of the degradation layer of pure magnesium *via* 3D imaging at resolutions below 40 nm

**DOI:** 10.1016/j.bioactmat.2021.04.009

**Published:** 2021-04-30

**Authors:** Berit Zeller-Plumhoff, Daniel Laipple, Hanna Slominska, Kamila Iskhakova, Elena Longo, Alexander Hermann, Silja Flenner, Imke Greving, Malte Storm, Regine Willumeit-Römer

**Affiliations:** a Helmholtz-Zentrum hereon GmbH, Institute of Metallic Biomaterials, Max-Planck-Straße 1, 21502, Geesthacht, Germany; b Helmholtz-Zentrum hereon GmbH, Research Reactor, Max-Planck-Straße 1, 21502, Geesthacht, Germany; c Helmholtz-Zentrum hereon GmbH, Institute of Materials Physics, Max-Planck-Straße 1, 21502, Geesthacht, Germany; d Helmholtz-Zentrum hereon GmbH, Institute of Materials Systems Modelling, Max-Planck-Straße 1, 21502, Geesthacht, Germany; e Diamond Light Source Ltd., Diamond House, Harwell Science and Innovation Campus, Didcot, OX11 0DE, United Kingdom

**Keywords:** Magnesium degradation, Porosity, Transmission X-ray microscopy, 3D imaging

## Abstract

Magnesium is attractive for the application as a temporary bone implant due to its inherent biodegradability, non-toxicity and suitable mechanical properties. The degradation process of magnesium in physiological environments is complex and is thought to be a diffusion-limited transport problem. We use a multi-scale imaging approach using micro computed tomography and transmission X-ray microscopy (TXM) at resolutions below 40 nm. Thus, we are able to evaluate the nanoporosity of the degradation layer and infer its impact on the degradation process of pure magnesium in two physiological solutions. Magnesium samples were degraded in simulated body fluid (SBF) or Dulbecco's modified Eagle's medium (DMEM) with 10% fetal bovine serum (FBS) for one to four weeks. TXM reveals the three-dimensional interconnected pore network within the degradation layer for both solutions. The pore network morphology and degradation layer composition are similar for all samples. By contrast, the degradation layer thickness in samples degraded in SBF was significantly higher and more inhomogeneous than in DMEM+10%FBS. Distinct features could be observed within the degradation layer of samples degraded in SBF, suggesting the formation of microgalvanic cells, which are not present in samples degraded in DMEM+10%FBS. The results suggest that the nanoporosity of the degradation layer and the resulting ion diffusion processes therein have a limited influence on the overall degradation process. This indicates that the influence of organic components on the dampening of the degradation rate by the suppression of microgalvanic degradation is much greater in the present study.

## Highlights

• The 3D nanoporosity of the degradation layer of magnesium is revealed using transmission X-ray microscopy.
• The degradation layer of magnesium contains a connected porous network.
• The porosity of the degradation layer has less influence on the degradation process than microgalvanic cells.

## 1 Introduction

Magnesium (Mg) alloys are highly attractive for their application as temporary bone implants, due to their mechanical properties, biocompatibility and biodegradability [1]. State-of-the-art titanium implants require removal after bone healing, as their permanent presence can lead to wear corrosion, fibrous encapsulation or inflammation [2]. By contrast, the degradation capability of Mg implants renders a second surgical intervention for removal of the implant in temporary bone healing applications obsolete. However, when Mg implants degrade too quickly, implant stability can be affected and a change in the local chemical environment can lead to cell death [[3], [4], [5]]. Therefore, the degradation rate of Mg implants must be carefully and reliably tailored for their ultimate application *in vivo*. To this end, *in vitro* experiments are required to single out different sub-processes for better understanding. For such *in vitro* experiments, different immersion media may be used, namely simulated body fluid (SBF), which exists in different compositions [[6], [7], [8]], Hank's balanced salt solutions or cell culture media, such as Dulbecco's Modified Eagle's Medium (DMEM) [9]. The ionic composition of these media, as well as the presence of proteins, e.g. through the addition of fetal bovine serum (FBS), has been shown to strongly influence the degradation process [[10], [11], [12], [13]]. It has been shown in terms of ionic components, that the interplay between HPO_4_^−^, HCO_3_^−^, Ca^2+^ and Cl^−^ ions is critical for the formation of a stable protective degradation layer [12,14,15]. If proteins are added to the immersion medium, they often lead to a change in degradation rate. Whether they increase or decrease the degradation rate depends partly on the immersion time [10,16,17] and on the alloy [18]. This is thought to be due to differing effects of chelation and adsorption. Chelation leads the binding of Mg^2+^ ions to proteins, in particular bovine serum albumin (BSA), and has been hypothesized to lead to an increase in degradation [16] and overall change in degradation mechanisms [17,19]. Protein adsorption is thought to play a major role in increasing corrosion resistance [20] and suppressing microgalvanic degradation in particular [16,21] by forming a protective layer on the sample surface. Apart from proteins, other organic components such as amino acids and glucose can have a strong influence on Mg degradation [10,22]. Iron (Fe)-complexing agents, such as folic acid or glucose, in particular can alleviate the re-depositioning of iron on Mg, which otherwise leads to continuous micro-galvanic corrosion [[23], [24], [25]]. A weak corrosion-inhibitory effects for a number of small organic components present in DMEM has also been demonstrated [26].

In addition to *in vitro* and *in vivo* modelling of Mg degradation, an increasing effort is placed into the development of computational models of the degradation processes in order to reduce the number of experiments that need to be carried out [27]. Many of the existing computational models include the transport of ions across the forming degradation layer (also often named a protective film), which is modelled as a porous medium [28,29]. This type of model assumes that the degradation process is mostly governed by the transport processes and will thus be diffusion-limited in a static environment. However, the porosity and tortuosity of the degradation layer, which determine the effective diffusion coefficient within, are mostly unknown and is therefore either fitted in the modelling process or obtained from 2D electron microscopy images.

Therefore, in order to design an accurate mechanistic and computational model of the degradation of Mg-based implants, it is necessary to image the porosity of the degradation layer in 3D, and to evaluate its connectivity and morphology at the highest possible resolution. The pore sizes in the degradation layer have previously been shown to be on the sub-micron scale [30,31], thus requiring resolutions of 100 nm and below. Therefore, we have employed full-field synchrotron-based transmission X-ray microscopy (TXM) tomography for the three-dimensional analysis of the nanoporous degradation layer morphology. Full-field TXM is a high-resolution imaging technique that requires X-ray condenser optics, namely Fresnel zone plates (FZPs) or Kirkpatrick–Baez (KB) mirrors to illuminate the sample and another set of FZPs for the focussing of the image onto the detector [32]. State-of-the art TXMs can currently reach resolutions down to 50 nm [33] and the usage of Zernike phase contrast enables the visualisation of low attenuating features [34]. TXM has previously been used successfully to image the time-lapse corrosion of Aluminium alloys [35].

In the present study, we have utilised full-field TXM with Zernike phase contrast at half-period resolutions below 40 nm to image the degradation layer of biodegradable Mg specimens. Thus, we are able to determine the three-dimensional morphology of the pore network within the degradation layer. Two commonly used immersion media were used as exemplary cases, namely SBF in the formulation presented by Bohner et al. [6] and DMEM, supplemented with 10% FBS (in the following abbreviated as DMEM+10%FBS). Both media are similar in ionic composition, see Supplementary Table 1, but DMEM+10%FBS is enriched with organic components and proteins, thus yielding a higher complexity. The immersion was performed over one to four weeks, to study whether a change in the pore network morphology takes place. Prior to TXM measurements, the degradation layer morphology was studied using micro computed tomography (μCT). The layer's crystalline composition was subsequently studied using X-ray diffraction (XRD). Similarly, scanning electron microscopy and energy dispersive X-ray spectroscopy (SEM + EDX) was used to analyse the chemical composition of the degradation layer. In this work, we demonstrate the large impact the presence of organic components has on the process of degradation in terms of degradation rate and morphology. We also show that the structure and nanoporosity of the degradation layer is however not influenced by the presence of proteins.

## 2 Materials and methods

Pure Mg (>99.92%) was cast and extruded at an extrusion ram speed of 2.4 mm/s as described by Wiese et al. [36]. The chemical impurities of the material were determined using spark spectrometry and revealed contents of 0.0048 ± 0.0001 wt% Fe, 0.0003 ± 0.0001 wt% Cu, <0.0002 wt% Ni, 0.0198 ± 0.0061 wt% Al and 0.0132 ± 0.0038 wt% Si. Discs (Ø = 9 mm, h = 1.5 mm) were cut from the ingots and shortly grinded using a 4000-grit paper to remove metallic residues from the cutting procedure. The discs were then cleaned and sterilized in a series of n-hexane (15 min), acetone (15 min), 100% (3 min) and 70% ethanol (15 min) in an ultrasonic bath. Subsequently, the discs were immersed in 3 ml of either SBF [6] or DMEM + Glutamax ((+) 4.5 g/L d-Glucose, (+) Pyruvate, Life Technologies, Darmstadt, Germany) supplemented with 10% FBS (Fetal Bovine Serum Superior, Sigma Aldrich, Merck, Darmstadt, Germany) and 1% Penicillin Streptomycin. The immersion time was set to 1, 2, 3 and 4 weeks and the samples were kept in an incubator to ensure physiological conditions (37 °C, 5% CO_2_). One disc was degraded per medium and time point. The immersion medium was changed every 3–4 days. At these time points, the medium pH and osmolality were measured using an SI line pH meter with MiniFET electrode (Sentron Europe, Leek Netherlands) and the Osmomat auto (Gonotec GmbH, Berlin, Germany). Following the immersion test, the samples were rinsed in double distilled water followed by 100% ethanol and stored in vacuum. Regions of interest of each sample were imaged using μCT at the Diamond Manchester Imaging Branchline I13-2 Diamond Light Source [37] to determine the overall degradation layer morphology. A pink beam with a mean energy of 29.1 keV was selected for imaging with a pco.edge 5.5 camera (PCO AG, Kelheim, Germany) at a magnification of 4×, resulting in a voxel size of 0.8125 μm, for all except two samples, which were imaged at 2× magnification, i.e. 1.625 μm voxel size. A detailed list of imaging parameters per sample can be found in Table 1. The μCT image data were reconstructed using the open-source Savu framework [38] with the TomoPy reconstruction package [39]. The images were binned twice and filtered using a 2D median filter with 3x3 kernel in Fiji/ImageJ [40]. The segmentation of the degradation layer was performed using the trainable WEKA plugin [41] with subsequent manual corrections in Avizo 2020.2 (FEI SAS, Thermo Scientific, France). The segmented image stacks were aligned such that the sample surface lay within the xy-plane of the images. The degradation layer depth was then computed using Matlab R2018a (The MathWorks Inc., USA) by measuring the number of voxels in z between the sample surface and the residual metal. This calculation was performed in the largest rectangular region fitting in to the aligned image without loss of information. Supplementary Fig. 1.

**Table 1:** Imaging settings for both micro computed tomography and transmission X-ray microscopy measurements for each sample.

		SBF				DMEM+10%FBS			
		*1w*	*2w*	*3w*	*4w*	*1w*	*2w*	*3w*	*4w*
*μCT*	Beamline	I13-2 (Diamond Light Source)							
	Energy	Pink beam (~29 keV)							
	Voxel size (μm)	0.8125	0.8125	1.625	0.8125	0.8125	0.8125	1.625	0.8125
*TXM*	Beamline	P05 (Petra III, DESY)		I13-2 (Diamond Light Source)		P05 (Petra III, DESY)		I13-2 (Diamond Light Source)	
	Energy (keV)	11.1		12		11.1		12	
	Voxel size (nm)	11.2		34.7		11.2		34.7	
	FOV (μm)	23		52.1		23		52.1	

For TXM small samples of approximately 25 μm diameter were cut from the degraded discs with one TXM sample per medium and time point. For this the crossbeam microscope Auriga from Zeiss was used, equipped with a Ga + focussed ion beam. The region to cut the TXM samples was selected randomly in the centre of the discs. Regions with large surface cracks were avoided to ensure the stability of the sample. The sample cylinders were milled by 10 nA currents and lifted out and transferred to mounting pins by a micromanipulator assisted by a gas injection system utilising tungsten. The 3- and 4-week samples were imaged using the TXM setup at I13-2 at Diamond Light Source [42]. Using a double multilayer monochromator (Mo/B4C coating, ΔE/E = 0.27%) an energy of E = 12 keV was selected. A beam shaping condenser with square fields [43] was used for sample illumination and the objective lens was an FZP with an outermost zone width dr = 50 nm. To enable phase contrast imaging, Zernike phase rings were installed in the back-focal plane of the FZP. 1- and 2-week samples were imaged at the nanotomography-endstation of the imaging beamline P05 operated by Helmholtz-Zentrum Geesthacht at PETRA III, DESY, at 11.1 keV using a Si111 double crystal monochromator (ΔE/E ~ 10^−4^), with a similar TXM setup as I13-2. The outermost zone width for the used FZP here was 30 nm, with corresponding condenser optics and phase rings. All optics were designed and fabricated in the X-ray Optics and Applications group of Paul-Scherrer-Institut (Switzerland).

Table 1 gives an overview of the applied imaging settings for each sample, including the beamline at which imaging was performed, the selected energy and voxel size, and the resulting field of view (FOV).

A Hamamatsu C12849–101U camera with a sCMOS chip with 6.5 μm physical pixel size and a 10 μm Gadox scintillation layer was used as detector system at both beamlines. At I13-2, the effective pixel/voxel size was 34.7 nm and a half-period resolution of approx. 100 nm was achieved. At P05, a voxel size of 11.2 nm could be reached, yielding a half-period resolution below 40 nm (Supplementary Fig. 2).

The samples were reconstructed using respective TomoPy pipelines [39] and filtered using an iterative non-local means filter [44]. Subsequently, the segmentation of the pores was performed using the trainable WEKA plugin, while the segmentation of the degradation layer and sample outline was performed in Avizo using manual segmentation and interpolation. For the analysis of the samples imaged at P05, only a circular region of the middle of the image stack was analysed to avoid the artefacts from region-of-interest tomography. For all samples, the uppermost 1–2 μm of the sample was removed, due to a loss of contrast (see Supplementary Fig. 3). The 3D histogram of the segmented porous degradation layer was computed in Fiji to calculate the overall porosity. Avizo was used to calculate the connected components of the pore network for the connectivity calculation. The connectivity of the network is defined as the ratio between the volume of the largest pore and the overall pore volume. At 100%, the network is fully connected. The tortuosity of the pore network was determined after skeletonization using the BoneJ plugin [45] in Fiji. The same plugin was used to determine the average and maximum pore size for each network.

The discs, from which TXM samples were cut, were subsequently measured used laboratory X-ray diffraction (XRD) and scanning electron microscopy (SEM) with energy dispersive X-ray spectroscopy (EDX) to enable a comparison of degradation products and resulting degradation layer porosity. XRD measurements were performed using a Cu K-α source (SEIFERT 3003 PTS, GE Inspection Technologies, Boston, US) operating at a wavelength of λ = 1.54 Å. A Meteor 1D detector was used with an exposure time of 10 min per 5° and a step size of 0.01°, i.e. 500 data points per 10 min, covering an overall 2θ range from 20° to 80°. The angle scale was calibrated for the three main Mg peaks with positions according to Downs et al. [46]. A non-degraded disc was measured as a reference.

The samples were subsequently embedded, grinded and polished to reveal a cross-section for SEM + EDX measurements. A Vega SB-U III (Tescan GmbH, Dortmund, Germany) with attached EDX unit was used for SEM + EDX measurements. SEM images and EDX maps were obtained at a magnification of 3000 for samples immersed in DMEM+10%FBS and at a magnification of 1250 for samples immersed in SBF. The magnification was chosen such that all samples of the same medium were imaged at the highest magnification for which the largest degradation layer still fit into the field of view. The acceleration voltage was set to 15 kV and the beam intensity to 18 for EDX measurements and 13 for SEM images. Based on the formulations of the degradation media, magnesium (Mg), sodium (Na), chloride (Cl), calcium (Ca), carbon (C), oxygen (O) and phosphorus (P) were investigated, and potassium (K) and sulphur (S) were also investigated in samples immersed in DMEM+10%FBS. The images were then analysed in terms of mean elemental weight percentage (wt%) as a function of distance to the metallic Mg, as described in more detail in a recent publication [12]. This analysis was performed in Matlab R2018a. Supplementary Fig. 4 shows a compilation of all SEM images and the chosen regions for EDX measurements.

All shown data plots were subsequently generated in Jupyter Notebooks from the Anaconda 3 package [47].

## 3 Results and discussion

### 3.1 Degradation layer thickness

**Fig. 1:**
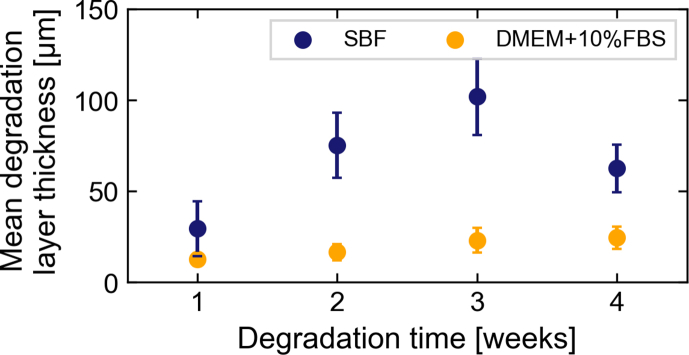
Mean degradation layer depth of the degraded pure Mg discs computed from μCT images. Blue markers show the results for samples immersed in simulated body fluid (SBF), while orange markers correspond to samples immersed in Dulbecco's Modified Eagles Medium (DMEM) supplemented with 10% fetal bovine serum (FBS). The standard deviation indicates the degradation homogeneity within the studied ROI. (For interpretation of the references to colour in this figure legend, the reader is referred to the web version of this article.)

The mean thickness of the degradation layer as determined using μCT, is given in Fig. 1. The degradation layer thickness for samples immersed in both media increases with time. Samples immersed in SBF show a significantly higher degradation depth than those degraded in DMEM+10%FBS. As the degradation layer depth for both media has been shown to equate to the degradation rate [12,48], this indicates a faster degradation in SBF. This may be due to the presence of proteins in DMEM+10%FBS in particular, as previously shown [10]. The standard deviation in Fig. 1 indicates the homogeneity of the degradation within the studied region-of-interest (ROI). It is notable that discs degraded in SBF display a significantly more inhomogeneous degradation behaviour than those in DMEM+10%FBS.

The sample degraded in SBF for four weeks shows a distinct deviation in degradation layer thickness. This may be due to the thick layer becoming instable and partially dissolving or breaking off during immersion testing. Apart from this sample, the degradation depths of samples immersed in SBF agree well with those found elsewhere [12] and unpublished work (Supplementary Fig. 5). The degradation layer thickness observed for samples immersed in DMEM+10%FBS agrees well with degradation rates and resulting mean degradation depths published for pure Mg in DMEM+10%FBS after one and four weeks [4].

**Fig. 2:**
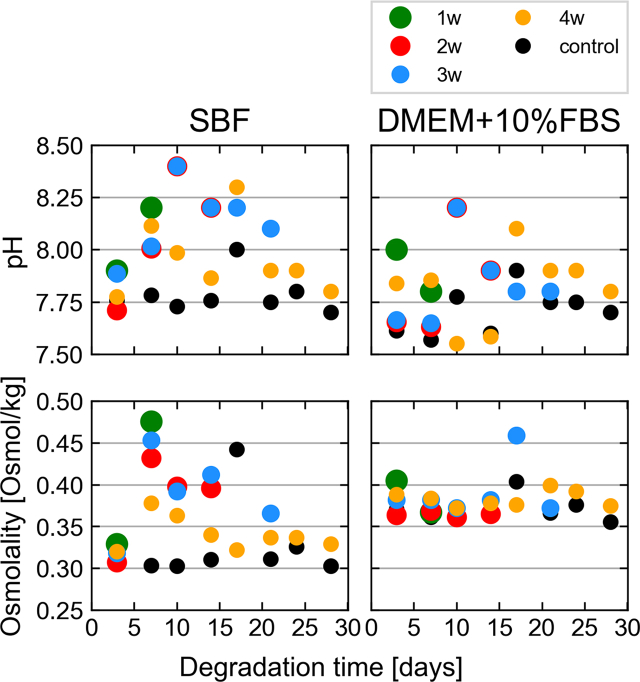
Evolution of pH and osmolality at time points of media change. The marker sizes and colors are varied to better display overlaying data points. The control data displays the mean of all control measurements at each time point. (For interpretation of the references to colour in this figure legend, the reader is referred to the web version of this article.)

During immersion testing, the media are refreshed every 3–4 days to avoid saturation with ions or degradation products. At these time points, the pH and osmolality of the degraded solution (and control solutions) are measured. The evolution in solution pH and osmolality corroborate findings from μCT measurements (Fig. 2).

Notably, the change of osmolality remains small for almost all time points for DMEM+10%FBS, while that for SBF is markedly higher. This suggests a faster degradation in SBF. The strong increase in osmolality in SBF between day 3 and day 7 highlights an acceleration in degradation after the first days. Additionally, chelation of Mg^2+^ ions released during degradation in DMEM+10%FBS may have contributed to the low change in osmolality. Similarly, changes in pH are higher in SBF than DMEM+10%FBS, except for week 1. Overall, the bulk pH changes are low, due to the environmental 5% CO_2_ in the incubator, which leads to pH buffering in the presence of HCO^3−^ ions in both media. It is notable, that the change in osmolality and pH for the sample immersed in SBF over 4 weeks appears lower than that of the other samples immersed in SBF.

### 3.2 Degradation layer composition

**Fig. 3:**
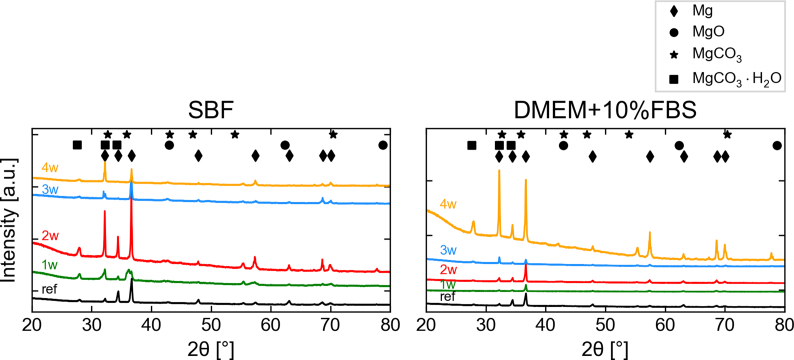
X-ray diffraction spectra of Mg discs degraded in SBF and DMEM+10%FBS obtained in a Cu K-α laboratory source. The identified compounds are indicated with respective markers as shown in the figure legend. The different XRD spectra are offset vertically for better visibility.

Spectra from laboratory XRD measurements for samples degraded in SBF and DMEM+10%FBS are given in Fig. 3. The measurements were focussed on the degradation layer but included bulk metal as well. A peak analysis shows that the same compounds are present in both media and for all time point, suggesting no change in the crystal structure of the degradation layer over time. Only Mg-containing compounds could be identified, namely Mg, MgO, MgCO_3_·H_2_O and MgCO_3_. The Mg peaks are more prominent in samples immersed in DMEM+10%FBS, which is likely to be due to the smaller overall depth of the degradation layer.

**Fig. 4:**
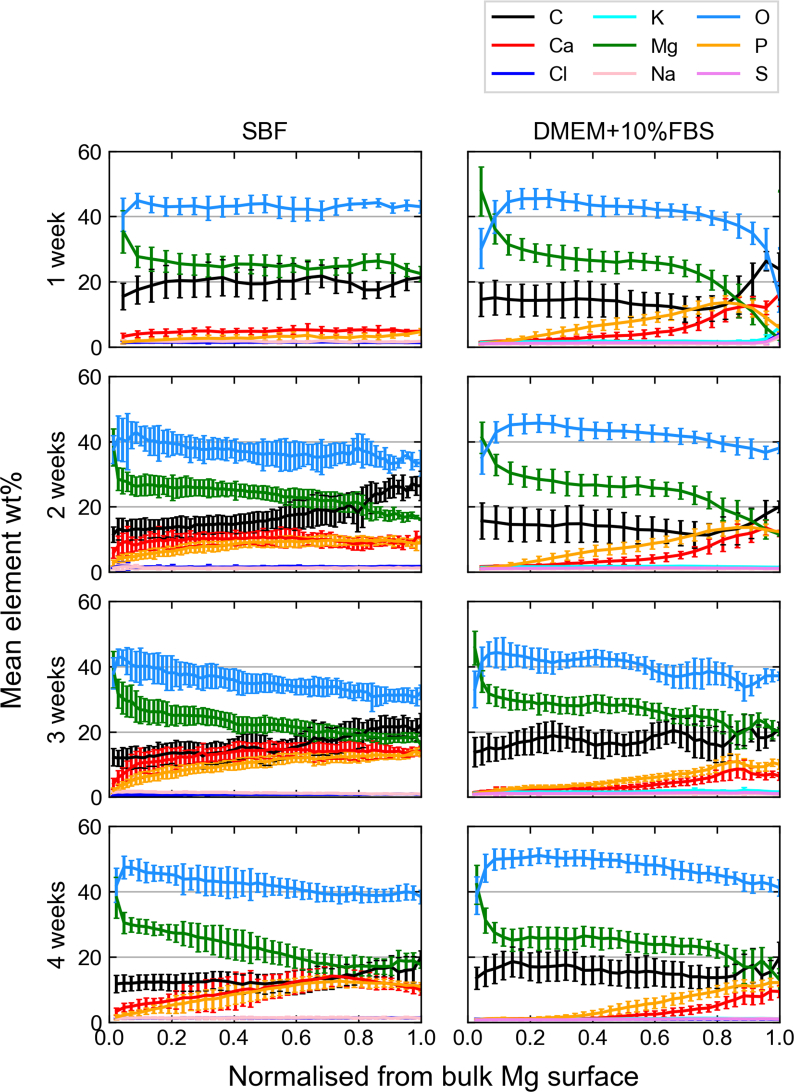
Elemental composition of the degradation layer as a function of distance to the metallic Mg surface as measured using EDX. The x-axis has been normalized to the degradation layer thickness for better comparison. The different colors indicate different elements as displayed in the figure legend. Ca and P precipitates increase towards the outside of the samples, while Mg, O and C are present throughout and more prominent towards the bulk metal. (For interpretation of the references to colour in this figure legend, the reader is referred to the web version of this article.)

EDX measurements corroborate the findings from the XRD analysis, based on the strong and coinciding C and Mg signals in the degradation layer (Fig. 4). Moreover, EDX data suggest the additional formation of (amorphous) Ca- and P-containing precipitates. These precipitates are more prominent towards the surface of the degradation layer and appear to increase relatively to Mg-containing precipitates over time. Samples immersed in SBF display a more homogeneous distribution of Ca and P in the degradation layer. The formation of Ca- and P-rich phases towards the outside of the degradation layer is in contrast to some findings [13,17], but in agreement with others [12,20,30,31]. Overall, XRD and EDX data suggest no significant structural differences between the degradation layers of pure Mg samples immersed in SBF and DMEM+10%FBS.

### 3.3 Degradation layer nanoporosity

**Fig. 5:**
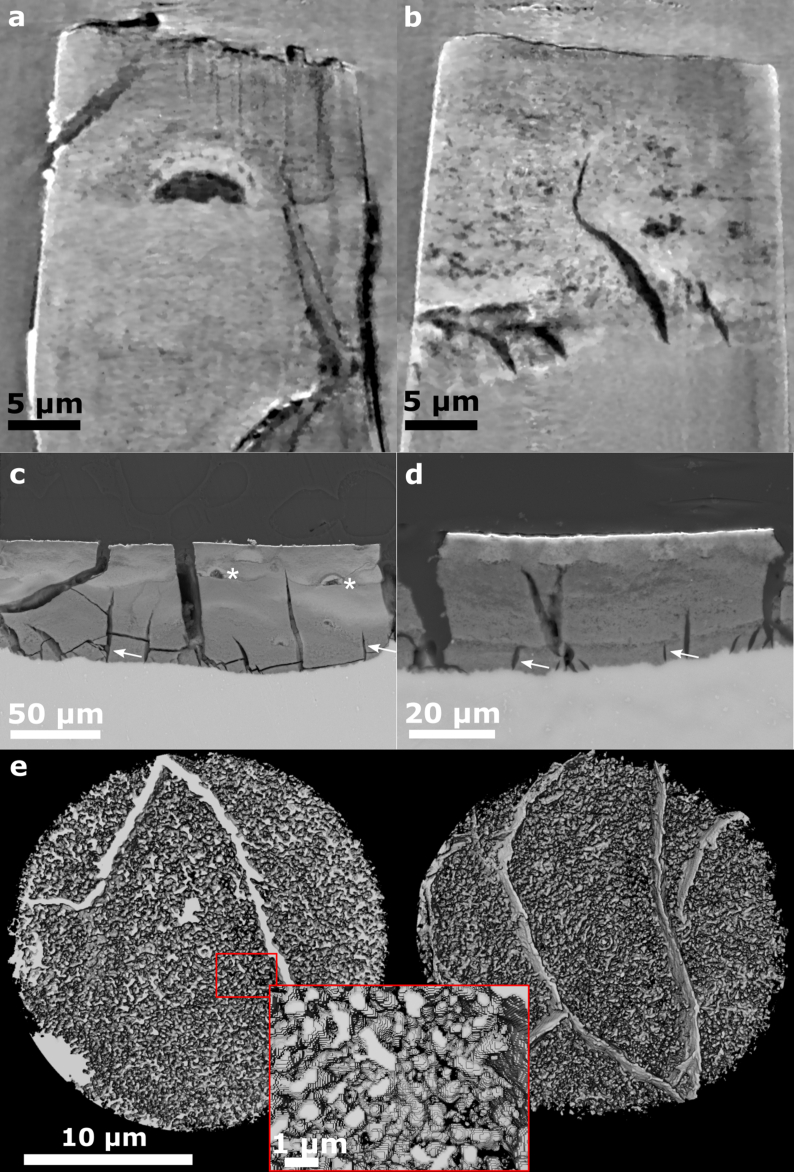
Representative slices through a TXM image volume of a sample immersed in SBF (a) and DMEM+10%FBS (b) for four weeks. The displayed images are after application of the iterative non-local means filter. Corresponding SEM images of cross-sections of the degraded Mg discs are shown in (c) and (d), respectively. (e) shows a rendering of the segmented pore network as seen from the top (left) and bottom (right), of the sample immersed in DMEM+10%FBS after 2 weeks and an inset with a zoom into the region displayed in red for better visibility of the pore sizes. For (a) only degradation layer is visible, however, (b–d) show both degradation layer and residual metal towards the bottom of the image. Small cracks are visible in the degradation layer of all samples (white arrows) and small pores can be seen within. The degradation layer of samples immersed in SBF also displays larger pores appearing as half-spheres surrounded by denser material (indicated by *). (For interpretation of the references to colour in this figure legend, the reader is referred to the web version of this article.)

Fig. 5 shows exemplary slices from TXM images of a sample immersed in SBF and DMEM+10%FBS after four weeks respectively (Fig. 5 a, b). For comparison, corresponding SEM cross-section images of the degradation layer of the bulk sample after preparation by grinding and polishing are shown (Fig. 5 c, d). Additionally, a 3D rendering of the segmented pore network is shown for the sample after immersion in DMEM+10%FBS after 2 weeks (Fig. 5 e).

Both SEM and TXM images show similar sub-micron morphologies of the degradation layer. Specifically, small cracks are visible in both image types, as well as smaller pores. Towards the bottom of Fig. 5 b-d residual Mg is visible, so that the interface between bulk material and degradation layer is revealed. The cracks appear to originate from the metal interface and smaller cracks are not reaching the degradation layer surface. The larger cracks visible in SEM images are likely to be drying artefacts, appearing only after the end of the degradation process. Whether the same holds true for the smaller cracks is not clear and can only be verified by *in situ* imaging in the future. As seen in the 3D rendering, the smaller pores form an interconnected network and appear to be connected to the smaller cracks.

**Fig. 6:**
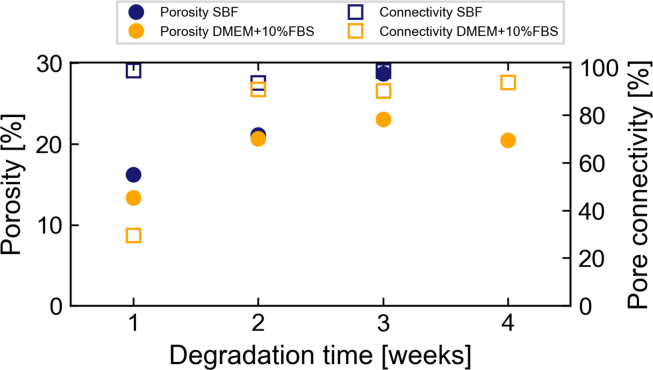
Measured porosity (circles) and pore connectivity (squares) based on TXM images of the pore network for pure Mg samples immersed in SBF (blue) and DMEM+10%FBS (orange). No value is shown for SBF after four weeks of immersion, as the pores were poorly visible in the lower part of the sample. (For interpretation of the references to colour in this figure legend, the reader is referred to the web version of this article.)

Fig. 6 shows that the overall nanoporosity and pore connectivity between all samples differ only slightly after 1 week of immersion. No values for SBF at 4 weeks are shown, as no pores could be segmented. This may be due to slight movements of the Zernike phase rings during imaging resulting in a strong loss of contrast. Fig. 6 shows that the pore connectivity for most samples is high (>90%), which highlights the interconnectivity of the pore network. The sample immersed in DMEM+10%FBS for 1 week however displays low pore connectivity (29.5%). As explained above, this is likely to be due to movement of the Zernike phase rings. Overall, the porosity determined for samples immersed in both media is similar and varies slightly around 20%.

In combination with XRD and SEM + EDX measurements, this suggests that similar degradation products form during the degradation of pure Mg in both media. These products appear to assemble in similar ways, as no differences in the networks’ tortuosity could be confirmed (Supplementary Fig. 6).

**Fig. 7:**
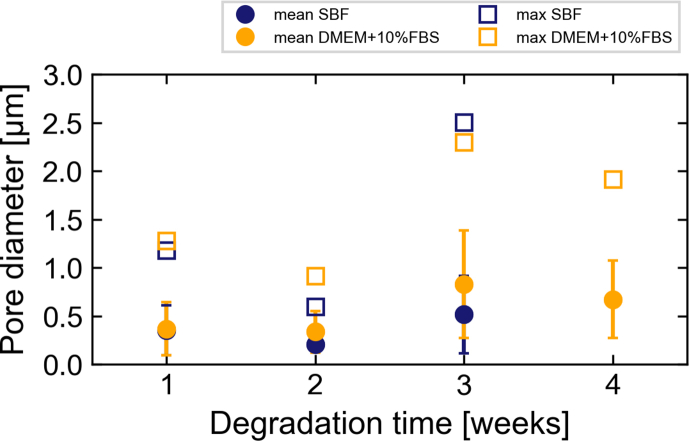
Mean (with standard deviation, circles) and maximum (squares) pore diameters for the pore networks in the degradation layer of samples immersed in SBF (blue) and DMEM+10%FBS (orange). No value is shown for SBF after four weeks of immersion, as the pores were poorly visible in the lower part of the sample. (For interpretation of the references to colour in this figure legend, the reader is referred to the web version of this article.)

Fig. 7 displays the mean and maximum pore diameter as determined for the pore network of each sample. The mean pore diameter for 1-week and 2-week samples ranges between 0.2 and 0.4 μm, while that of 3-week and 4-week samples is between 0.5 and 0.83 μm. The pore diameter will be strongly influenced by the cracks that are apparent in most samples and by the general image resolution. The pore sizes are generally large enough for ions to diffuse but also for larger molecules, such as proteins, to pass through.

**Fig. 8:**
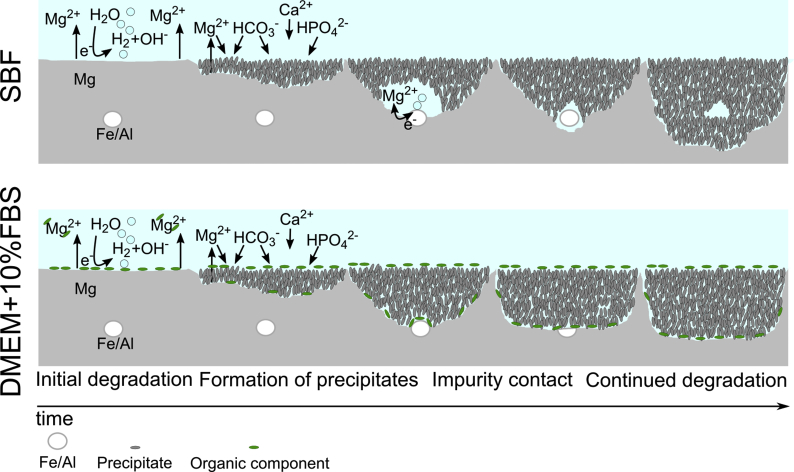
Schematic of the degradation process of pure Mg containing impurities in SBF and DMEM+10%FBS. The initial process of metal degradation and precipitate formation is similar in both media. In DMEM+10%FBS organic materials are building an additional protective layer. When the medium comes into contact with the metallic impurity, the process changes significantly. In SBF, a microgalvanic cell is developing leading to increased formation of hydrogen and degradation. A distinct volcano-shaped gap remains in the degradation layer around the former impurity. In DMEM+10%FBS however, the organic matter suppresses the evolution of the micro-galvanic cell and the degradation process remains unaltered. Not to scale.

While the nanoporosity of samples immersed in both media is similar, samples immersed in SBF demonstrate larger pores in the shape of half-spheres that are apparently surrounded by denser material (see Fig. 5 a, c). These features are not visible in samples immersed in DMEM+10%FBS. The TXM measurements of samples immersed in SBF for 3 and 4 weeks contained such half-spherical pore, which may explain the larger porosity calculated for the 3-week sample (Fig. 6). The occurrence of these distinct features can be explained by considering the degrading material: as the purity of the Mg discs used in the experiments was limited to approximately 99.92%, small metallic impurities are present in the material. These could also be visualised using TXM (Supplementary Fig. 3) as dense material clusters in the Mg matrix with an approximate size of 250–300 nm. Based on the sample material composition, these clusters are likely to be Fe- or Al-based. Therefore, we hypothesise that the observed areas of densification and larger pores are residues from previous microgalvanic cells. The shape of the pore is consistent with volcano-shapes previously reported for microgalvanic degradation of Mg-alloys and pure Mg in SBF [49]. These microgalvanic cells formed only in SBF and significantly accelerated the degradation process. Depending on their distribution within the Mg matrix, the degradation was accelerated locally, leading to the observed inhomogeneity of degradation layer thickness (Fig. 1). As the ionic composition of both immersion media is similar and the same base material was used, this could suggest that the presence of organic components in DMEM+10%FBS suppressed the evolution of these microgalvanic cells, thus controlling the speed and homogeneity of degradation. This process is displayed schematically in Fig. 8. Previous authors have attributed the more homogeneous degradation in protein-containing solutions to the fact that proteins adsorb to the surface of the material and fill pits formed from microgalvanic degradation [16]. Alternatively, it was suggested that small organic components such as folic acid prevent the re-depositioning of Fe [23,24], thereby inhibiting severe micro-galvanic corrosion. Based on the absence of the volcano-shaped pores in samples immersed in DMEM+10%FBS, we hypothesise that the proteins or organic components have adsorbed also to the impurity at the onset of microgalvanic corrosion, hindering their evolution altogether. Albumin for example was shown to adsorb preferably to aluminiumoxide rather than magnesiumoxide or hydroxyapatite [50], which strengthens the case for this scenario. Future *in situ* imaging of the degradation process will enable the direct proof of this microgalvanic cell suppression. Which organic component or protein in particular has led to the suppression of galvanic degradation cannot be determined based on this experiment. Penicillin-streptomycin, which was also supplemented to DMEM+10%FBS, might however be excluded, as it was previously shown to have little effect or accelerate Mg degradation, rather than decelerate it [24].

The present study shows that the nanoporosity of the degradation layer has only limited influence on the degradation process itself. The nanoporosity will influence the transport of ions between the degradation medium and the metallic surface, but in the absence of organic components, the degradation process of Mg in aqueous media cannot be considered as a diffusion limited problem. By contrast, the presence of organic components is a reason for the controlled degradation of Mg alloys *in vitro* and *in vivo*, potentially even if large intermetallic phases are present in the alloy. Similarly, detrimental effects such as iron re-depositioning, may be alleviated *in vivo.* This emphasises the fact that *in vitro* testing of biodegradable Mg implants should always be performed in more complex media. We expect that larger differences in the degradation layer morphology would be observed, if we studied different Mg alloys in the same degradation medium, in particular in DMEM+10%FBS. Due to the presence of alloying elements, the degradation layer composition and thus its morphology is expected to differ significantly for different alloys. Future experiments should focus on elucidating this dependence.

Despite the ability to visualise pores within the degradation layer, the resolution of TXM is still relatively low for studying the pore network morphology in detail and samples are prone to movement artefacts. Therefore, higher resolution 3D imaging using transmission electron tomography (TEM) should be performed in the future to verify the obtained pore network morphology. TEM enables highest resolution imaging down to the atomic scale and has previously been used e.g. to study dealloying of submicrometric intermetallic particles [30]. Thus, the degradation process can be understood in greater detail. A drawback to TEM are the smaller investigated volumes, as often thin sections are prepared using focussed ion beam milling with a maximum height of 10 μm. This would limit the degradation layer analysis to short time points and the thickness of the sections limits the depth resolution. TXM enables imaging of larger sample volumes at high resolution. While *in situ* imaging using TEM is possible, it is more challenging that *in situ* TXM measurements. Nevertheless, both techniques should be considered as complementary and they yield the most information on the overall degradation process if combined. Other complementary methods, such as atomic force microscopy, coupled with scanning electrochemical microscopy should be considered in the future to confirm galvanic coupling in the early stages of Mg degradation.

## 4 Conclusion

The present study provides the first proof that the degradation layer of biodegradable Mg contains an interconnected three-dimensional nanoporous network. This is an important finding for the mechanistic and computational modelling of the transport processes involved during the degradation. Utilising multiscale X-ray imaging by means of μCT and TXM, we have shown that the presence of organic components is critical for the controlled degradation of impurity-containing Mg. Specifically, organic components appear to suppress the evolution of microgalvanic cells, which would otherwise accelerate the degradation. TXM and corresponding measurements using XRD and SEM + EDX revealed no significant influence of the organic components on the nanoporosity, and structural and chemical composition of the degradation layer. A detailed analysis should take place to determine which organic component leads to the suppression of the microgalvanic cells and whether the findings can be translated to Mg alloys in general.

## Author contributions

The study was conceived and planned by BZP in discussion with RWR. Immersion tests and μCT experiments were performed by BZP, with support by MS at I13-2. Sample preparation for TXM was performed by DL. TXM experiments at I13-2 were performed by BZP, HS, AH and EL with support by MS, TXM measurements at P05 were performed by BZP with support by EL, SF and IG. SEM + EDX preparation was conducted by HS with imaging performed both by HS and BZP. XRD experiments and analysis was performed by KI. All other analysis was performed by BZP. The document was chiefly written by BZP with input from all parties.

## Funding sources

HS and KI acknowledge funding from the European Union's Horizon 2020 research and innovation program under the Marie Skłodowska-Curie grant, agreement No 811226. EL and IG gratefully acknowledge the financial support from the Deutsche Forschungsgemeinschaft (DFG, German Research Foundation)–Projektnummer 192346071, SFB 986, project Z2.

## CRediT authorship contribution statement

**Berit Zeller-Plumhoff:** Conceptualization, Investigation, Formal analysis, Visualization, Writing – original draft, Writing – review & editing. **Daniel Laipple:** Investigation, Writing – review & editing. **Hanna Slominska:** Investigation, Formal analysis. **Kamila Iskhakova:** Investigation, Formal analysis. **Elena Longo:** Investigation, Writing – review & editing. **Alexander Hermann:** Investigation. **Silja Flenner:** Investigation, Writing – review & editing. **Imke Greving:** Investigation, Writing – review & editing. **Malte Storm:** Investigation, Writing – review & editing. **Regine Willumeit-Römer:** Writing – review & editing.

## Declaration of competing interest

The authors declare that they have no known competing financial interests or personal relationships that could have appeared to influence the work reported in this paper.
