# Supplementary material for: Evaluating the morphology of the degradation layer of pure magnesium via 3D imaging at resolutions below 40 nm
Source: Bioact Mater. 2021 Apr 30;6(12):4368–76. doi: 10.1016/j.bioactmat.2021.04.009 (PMC8111030; doi:10.1016/j.bioactmat.2021.04.009)
Supplement: Multimedia component 1 [file mmc1.docx]

Supplementary material for “Evaluating the morphology of the degradation layer of pure magnesium *via* 3D imaging at resolutions below 40nm”

*Berit Zeller-Plumhoff^*^**^§^, Daniel Laipple^¥^, Hanna Slominska^§^, Kamila Iskhakova^§^, Elena Longo^†^, Alexander Hermann^‡^, Silja Flenner^†^, Imke Greving^†^, Malte Storm^£^, Regine Willumeit-Römer^§^*

*^§^ Helmholtz-Zentrum hereon GmbH, Institute of Metallic Biomaterials, Max-Planck-Straße 1, 21502 Geesthacht, Germany*

*^¥^ Helmholtz-Zentrum hereon GmbH, Research reactor, Max-Planck-Straße 1, 21502 Geesthacht, Germany*

*^†^ Helmholtz-Zentrum hereon GmbH, Institute of Materials Physics, Max-Planck-Straße 1, 21502 Geesthacht, Germany*

*^‡^ Helmholtz-Zentrum hereon GmbH, Institute of Materials Systems Modelling, Max-Planck-Straße 1, 21502 Geesthacht, Germany*

*^£^ Diamond Light Source Ltd., Diamond House, Harwell Science and Innovation Campus, Didcot, OX11 0DE, United Kingdom*


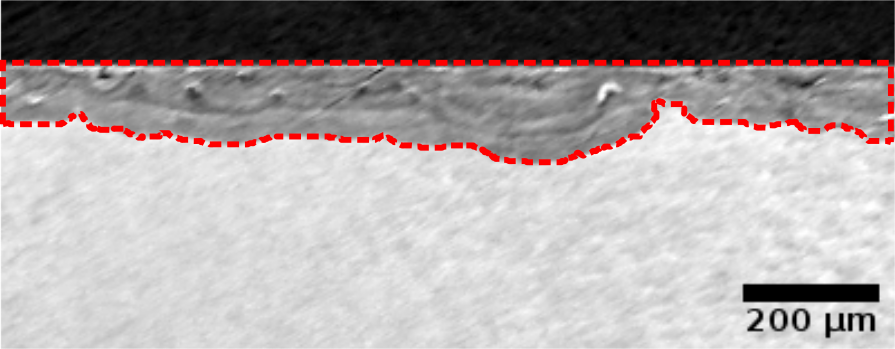


**Supplementary Figure 1.** Slice through tomographic reconstruction following µCT imaging of the sample immersed in SBF for 3 weeks. The degradation layer is clearly visible and is outlined. The inhomogeneity of the degradation layer thickness in this sample can be observed.


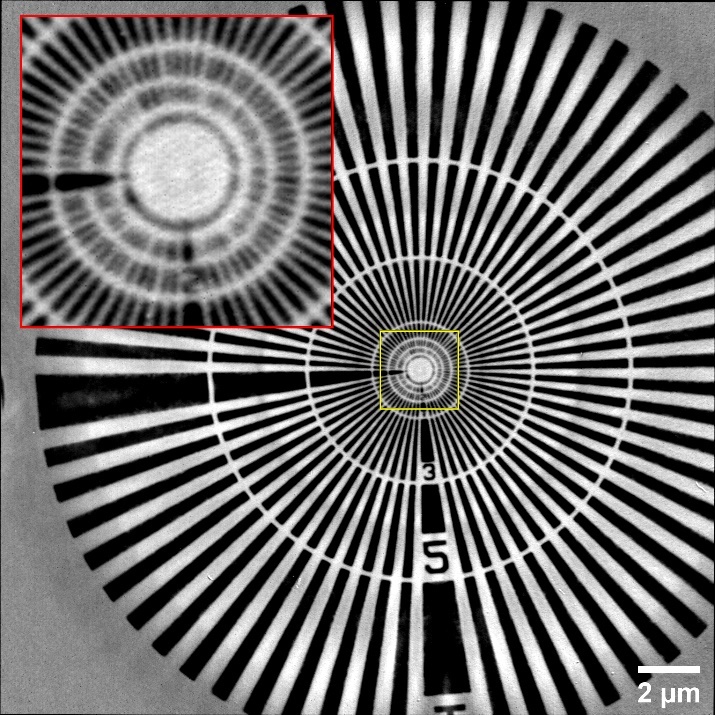


**Supplementary Figure 2.** Siemensstar with inlet displaying the 25-35 nm lines (2^nd^ ring from inside) and 37-48 nm lines (3^rd^ ring) obtained for TXM using Zernike phase contrast at P05, PETRAIII, DESY.


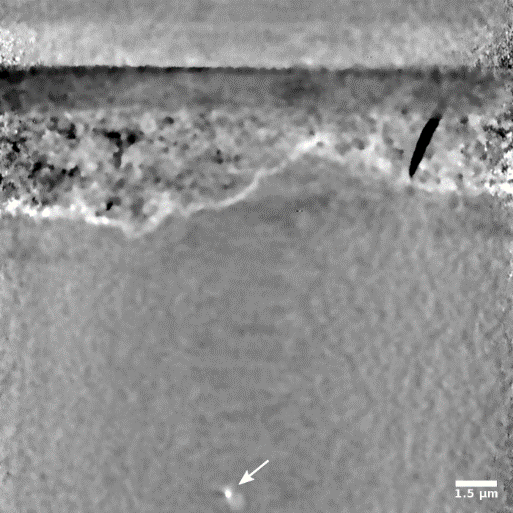


**Supplementary Figure 3.** Slice through the tomographic reconstruction of the sample immersed in DMEM+10%FBS for 1 week imaged using TXM. The scale bar is 1.5 µm. Small metallic particles are visible in the non-degraded part of the sample (white arrow). Image artefacts due to region-of-interest-scanning are visible at the borders of the image. These were cropped for image analysis. Similarly, the uppermost part of the sample cannot be analysed due to a strong loss of contrast from phase contrast imaging.


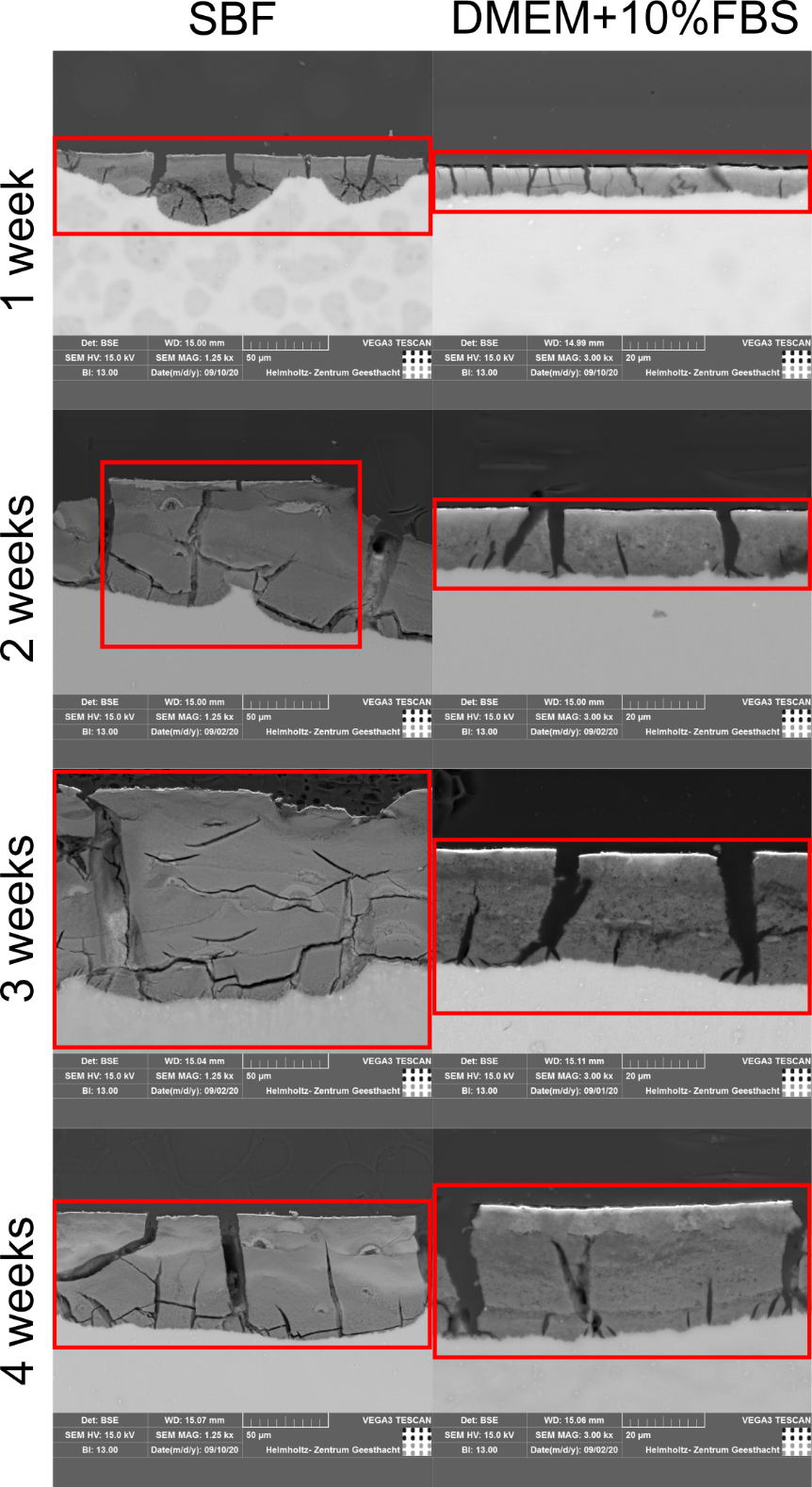


**Supplementary Figure 4.** Compilation of SEM images of cross-sections of all samples. The area selected for EDX measurements is shown in red. The scale bar for samples immersed in SBF is 50 µm, that for samples immersed in DMEM+10%FBS 20 µm.


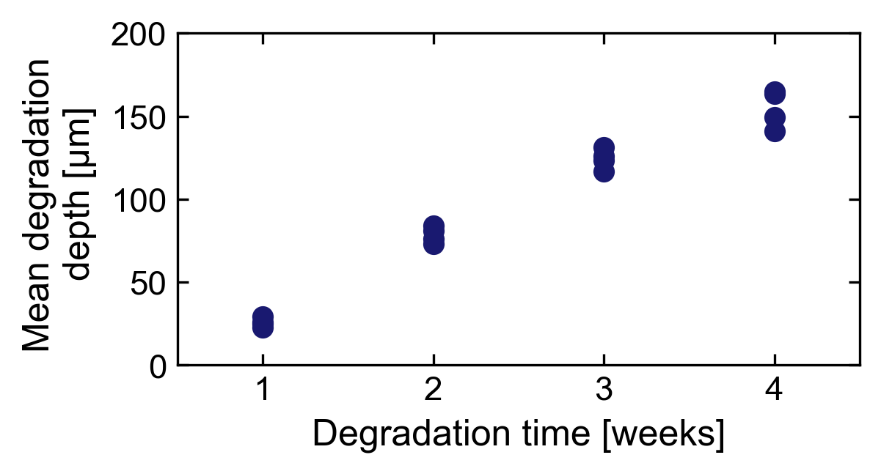


**Supplementary Figure 5.** Mean degradation depth as computed for samples (n=4 per time point) immersed in SBF over 1-4 weeks *via* weight loss measurements in a separate but identical experiment as described above.


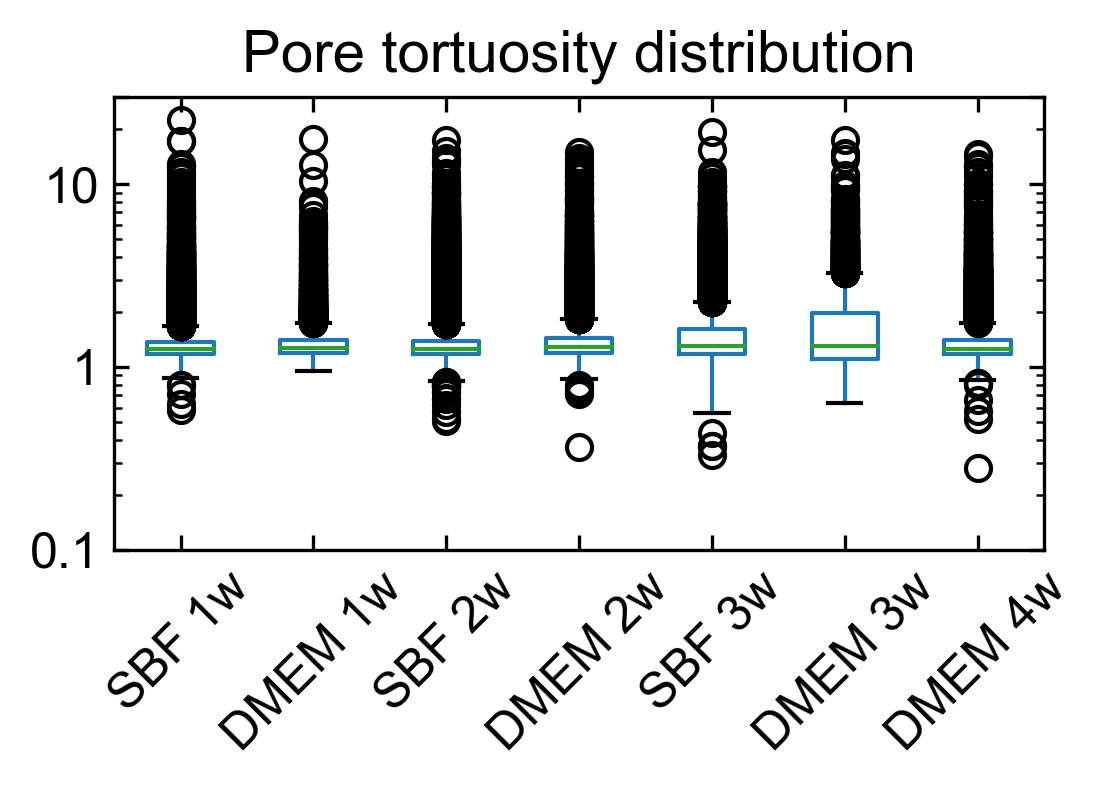


**Supplementary Figure 6.** Pore tortuosity distribution for all samples imaged using TXM. Strong outliers are visible due to the apparent cracks in the pore network. The majority of pores display small tortuosity values.

SUPPLEMENTARY TABLES

| Ingredient | Concentration in SBF [1] [mmol/l] | Concentration in DMEM [mmol/l] |
| --- | --- | --- |
| Na^+^ | 142.7 | 155.3 |
| K^+^ | 0 | 5.3 |
| Ca^2+^ | 2.43 | 1.8 |
| Mg^2+^ | 0 | 0.8 |
| Cl^-^ | 114.5 | 115.7 |
| HCO_3_^-^ | 35.1 | 44.1 |
| HPO_4_^2-^ | 1.4 | 0.9 |
| SO_4_^2-^ | 0 | 0.8 |
| Fe^3+^ | 0 | 2E-4 |
| NO_3_^-^ | 0 | 6E-4 |
| Amino acids | 0 | 10.6 |
| Vitamins | 0 | 0.15 |
| Glucose | 0 | 25 |
| Phenol red | 0 | 0.04 |

**Supplementary Table 1.** Composition of immersion media used in this study. DMEM was supplemented with 10%FBS and 1%Penicillin in this study.

REFERENCES

[1] M. Bohner, J. Lemaitre, Can bioactivity be tested in vitro with SBF solution?, Biomaterials. 30 (2009) 2175–2179. https://doi.org/10.1016/j.biomaterials.2009.01.008.
